# The Mycotoxin Deoxynivalenol Significantly Alters the Function and Metabolism of Bovine Kidney Epithelial Cells In Vitro

**DOI:** 10.3390/toxins11100554

**Published:** 2019-09-20

**Authors:** Jennifer R. Bailey, Jonathan Breton, Gordana Panic, Tristan A. Cogan, Michael Bailey, Jonathan R. Swann, Michael R. F. Lee

**Affiliations:** 1 Bristol Veterinary School, University of Bristol, Langford, Bristol BS40 5DU, UK; tristan.cogan@bristol.ac.uk (T.A.C.); mick.bailey@bristol.ac.uk (M.B.); michael.lee@rothamsted.ac.uk (M.R.F.L.); 2 Department of Metabolism, Digestion and Reproduction, Faculty of Medicine, Imperial College London, South Kensington Campus, London SW7 2AZ, UK; j.breton@imperial.ac.uk (J.B.); g.panic@imperial.ac.uk (G.P.); j.swann@imperial.ac.uk (J.R.S.); 3 Rothamsted Research, North Wyke, Okehampton, Devon EX20 2SB, UK

**Keywords:** mycotoxicosis, metabolic profiling, protein synthesis, cell cycle arrest

## Abstract

Bovine mycotoxicosis is a disorder caused by the ingestion of fungal toxins. It is associated with chronic signs, such as reduced growth rate and milk yield, and causes significant economic cost to the dairy industry. The mycotoxins deoxynivalenol (DON), zearalenone (ZEN), and fumonisin B1 (FB1) are commonly found in grain fed to cattle. Patulin (PA) is a common grass silage contaminant but is also found in grain. The effects of these mycotoxins on cellular function at low concentrations are not well understood. Using Madin–Darby bovine kidney cells we evaluated the cellular response to these mycotoxins, measuring cytotoxicity, de novo protein synthesis, cell proliferation, cell cycle analysis, and also metabolic profiling by ^1^H NMR spectroscopy. DON, ZEN, and PA induced cytotoxicity, and PA and FB1 induced a decrease in metabolic activity in surviving cells. DON was the only mycotoxin found to have a significant effect on the metabolic profile, with exposed cells showing increased cellular amino acids, lactate, 2-oxoglutarate, 3-hydroxybutyrate, and UDP-*N*-acetylglucosamine and decreased β-alanine, choline, creatine, taurine, and *myo*-inositol. Cells exposed to DON also showed reductions in protein synthesis. DON has previously been documented as being a ribotoxin; the results here suggest that exposure of bovine cells to DON causes a decrease in protein synthesis with corresponding cellular accumulation of precursors. Cell proliferation was also arrested without causing apoptosis. It is likely that exposure triggers hypoxic, hypertonic, and ribotoxic responses in bovine cells, and that these responses contribute to reduced productivity in exposed cattle.

## 1. Introduction

Bovine mycotoxicosis is a disorder most commonly caused by the ingestion of fungal toxins (mycotoxins) within feed. Though acute manifestations exist, bovine mycotoxicosis most frequently constitutes a range of chronic and non-specific symptoms including digestive problems, such as gastroenteritis, diarrhea, and depressed feed intake [1,2], reproductive problems such as ovarian cycle irregularities and infertility [3,4], immunosuppression [5,6], and neurological disorders [7,8]. This in turn can impact productivity through reduced growth rates and milk yield [9,10], which is thought to confer significant economic losses to the dairy industry [11,12,13]. The source of the problem comes predominately from mold-contaminated feed such as grain and forage silage, especially foraged maize (*Zea mays*). Silage (including whole crop (grain and straw), maize and grass) is the main conserved forage source for dairy and beef cattle, and represents a significant source of mycotoxins; yet comparatively little research has been conducted into the effects of ingestion of mycotoxin-contaminated silage on cattle [14]. An underlying reasoning for the skewed research activity when it comes to mycotoxins in livestock toward monogastrics (pigs and poultry) is the ability of ruminants to detoxify mycotoxins in the rumen [1]. For example, the mycotoxin deoxynivalenol (DON) is metabolized to de-epoxy derivatives and zearalenone (ZEN) to zearalanone and zearalenol metabolites [15]. However, the extent of detoxification depends on the dose rate, rumen digestion kinetics, and the microbial community [16]. In particular, high-production dairy cows are offered diets (cereals and grains rich in starch) that modify the microbial community structure of the rumen away from fibrolytic (fiber degrading) bacteria toward starch-degrading organisms. Ultimately, this reduces the pH of the rumen, altering the microbial ecosystem as the animal approaches acidosis, substantially reducing the ability of the rumen to detoxify mycotoxins [15]. 

A recent analysis of silage samples in southwestern England found 90% of maize samples were contaminated with common mycotoxins, with over 46% of those samples containing mycotoxin concentrations in the moderate-to-high risk categories [17,18]. The main causative agents are fungi of the *Fusarium, Aspergillus,* and *Penicillium* species, which together produce a range of toxins [19]. The ones most commonly associated with bovine sickness are aflatoxins (AF), ochratoxin (OTA), patulin (PA), fumonisin, particularly fumonisin B1 (FB1), and the tricothecenes ZEN and DON [1,20]. DON is the best known and most common contaminant of grains, and its detection in feed is commonly a marker for the presence of other toxins [21]. Recent surveys of raw feed samples globally found that 59% were contaminated with an average level of 1 mg/kg and a maximum level of 49 mg/kg of DON, suggesting a high likelihood of significant exposure across the life-course for most ruminants [22]. The effects of mycotoxins and their metabolites on several bovine cell and tissue types, such as their ruminal microbiota [23], monocytes [24], or ovarian cells [25,26], have been characterized and have contributed to our understanding of how their ingestion may cause the above-described pathologies. In this work, the impact of the commonly ingested mycotoxins, DON, ZEN, FB1, and PA, on a bovine kidney epithelial cell line (Madin–Darby bovine kidney; MDBK) were investigated. The effects of these mycotoxins on cellular division, protein synthesis, and metabolism were studied to gain insight on how these toxins might affect epithelial and specifically kidney epithelial cell function.

## 2. Results

### 2.1. DON and ZEN Are Cytotoxic at High Doses but Do Not Reduce Cell Metabolism at Lower Doses

**Figure 1:**
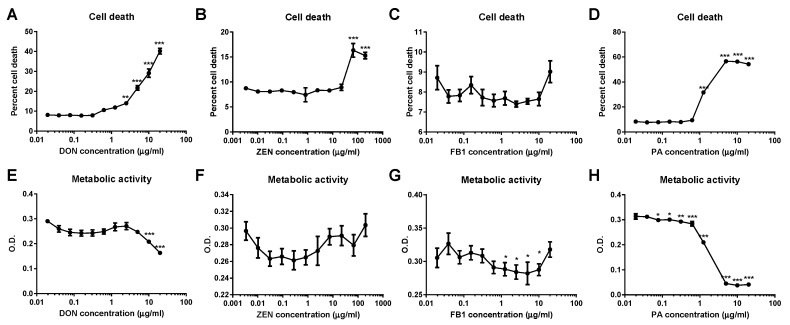
Cytotoxicity and metabolic activity of Madin–Darby bovine kidney (MDBK) cells following exposure to deoxynivalenol (DON), zearalenone (ZEN), fumonisin B1 (FB1), and patulin (PA). Percent cell death in MDBK populations treated with DON (**A**), ZEN (**B**), FB1 (**C**), or PA (**D**); cell death was significantly increased when compared to control populations when cells were exposed to DON, ZEN, and PA. Metabolic activity was significantly reduced following exposure to DON (**E**), FB1 (**G**), and PA (**H**) but not ZEN (**F**). Data are shown from four replicate experiments and are presented as mean ± standard error of the mean (SEM). * *p* < 0.05, ** *p* < 0.01, and *** *p* < 0.001. O.D. = optical density.

DON, ZEN, and PA all demonstrated a cytotoxic effect on MDBK renal epithelial cells, measured by the release of lactate dehydrogenase (LDH); cell death was significantly higher at concentrations of greater than or equal to 2.25 µg/mL DON, 67 μg/mL ZEN, and 1.25 μg/mL PA (Figure 1A,B,D). Exposure to FB1 had no effect on MDBK cell death (Figure 1C). Cells treated with FB1 or PA showed a significant reduction in metabolic activity, determined by thiazolyl blue tetrazolium bromide (MTT) assay, at mycotoxin concentrations below the level at which cytotoxicity was observed (Figure 1G,H). Conversely, no difference in metabolic activity was observed in MDBK cells treated with ZEN (Figure 1F) and a significant reduction in metabolic activity was only detected in MDBK cells treated with concentrations of DON of 10 µg/mL or higher (Figure 1E); at lower levels, DON and ZEN did not induce a decline in metabolic activity prior to reaching a concentration at which significant levels of cell death were observed.

### 2.2. DON Induces Metabolic Perturbations in MDBK Cells

**Figure 2:**
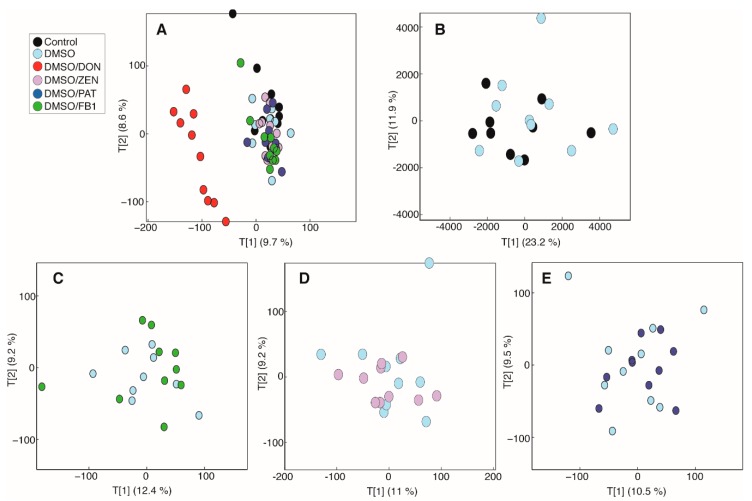
Principal-components analysis (PCA) models comparing the intracellular metabolic profiles of all cells from the study measured by ^1^H NMR spectroscopy. (**A**) Scores plot from the PCA model comparing the intracellular metabolic profiles from all cells (PC1 vs. PC2; R^2^ = 18.3%). Scores plots from pairwise PCA models comparing (**B**) control and DMSO-treated cells (PC1 vs. PC2; R^2^ = 35.1%), and DMSO-treated cells with those treated with (**C**) 0.5 µg/mL fumonisin B1/DMSO (PC1 vs. PC2; R^2^ = 21.6%) (FB1), (**D**) 20 µg/mL zearalenone (ZEN)/DMSO (PC1 vs. PC2; R^2^ = 20.2%), and (**E**) 0.1 µg/mL patulin (PA)/DMSO (PC1 vs. PC2; R^2^ = 20%).

To further characterize cellular metabolic perturbations induced by the above-tested mycotoxins, MDBK cells were cultured with mycotoxin concentrations below those observed to induce significant cytotoxicity (1 µg/mL DON, 20 µg/mL ZEN, 0.5 µg/mL FB1, and 0.1 µg/mL PA) and the cell lysate was analyzed by ^1^H NMR spectroscopy. A principal-components analysis (PCA) model was built comparing the biochemical profiles of all study groups. The scores plot from this model showed that DON treatment in 0.5% DMSO was driving the largest amount of variation in the metabolic data (Figure 2). DMSO alone was not found to impact on the metabolic landscape of MDBK cells (Figure 2B). At the tested doses, the other mycotoxins did not induce any metabolic alterations. This was confirmed by pairwise PCA models comparing the metabolic profiles of cells treated with different mycotoxins against those treated with DMSO (Figure 2C–E).

**Figure 3:**
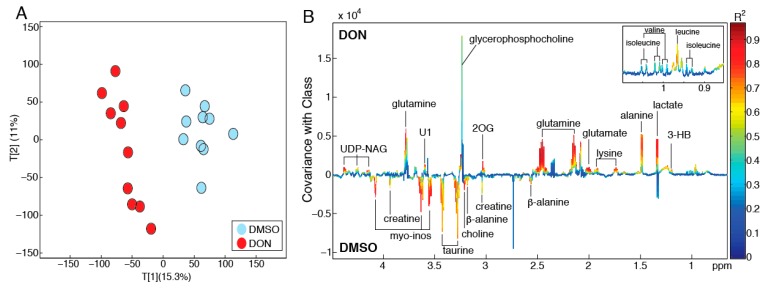
Biochemical perturbations induced by DON in MDBK cells. (**A**) PCA model constructed on the ^1^H NMR profiles of cell extracts obtained from MDBK cells treated with DMSO and DON/DMSO. Scores plot (PC1 vs. PC2; R^2^ = 26.3%) showing clear metabolic variation between the cells following exposure to DON. (**B**) Coefficients plot extracted from the partial least squares-discriminant analysis (PLS-DA) model comparing the intracellular metabolic profiles of DMSO and DON/DMSO-treated cells (Q^2^Y = 0.88; *p* = 0.001). 2OG = 2-oxoglutarate; 3-HB = 3-hydroxybutyrate; myo-inos = *myo*-inositol; UDP-NAG = UDP-*N*-acetylglucosamine; U1 = Unknown metabolite.

To further explore the metabolic derangements caused by DON exposure in MDBK cells, a pairwise PCA model was constructed comparing the intracellular metabolic profiles of cells treated with DMSO only with those treated with DON in DMSO (Figure 3A). The scores plot of this model shows clear separation between the two treatment groups in the first principal component (PC1, R^2^ = 15.3%). A partial least squares-discriminant analysis (PLS-DA) model was built on the same profiles to identify metabolic variation associated with DON exposure (Figure 3B). This PLS model had a strong predictive performance (Q^2^Y = 0.88; *p* = 0.001) and DON exposure was observed to increase cellular amino acids (alanine, glutamate, glutamine, isoleucine, leucine, valine, tyrosine), glycerophosphocholine, lactate, 2-oxoglutarate, 3-hydroxybutyrate, and UDP-*N*-acetylglucosamine and decrease β-alanine, choline, creatine, taurine, and *myo*-inositol.

### 2.3. MDBK Cells Exposed to DON Showed Reduced Levels of Protein Synthesis

**Figure 4:**
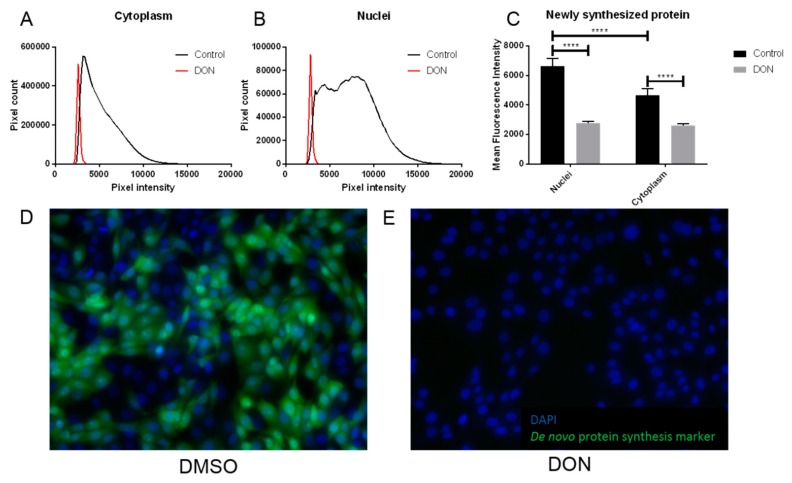
Cells treated with DON showed significantly reduced levels of protein synthesis compared to controls. De novo protein synthesis was detected in populations of MDBK cells exposed to DON/DMSO or DMSO (control) by production of a green signal, visible by immunohistology. The intensity of this signal in cytoplasm (**A**) and nuclei (**B**) was quantified, demonstrating that treatment with DON/DMSO reduced this signal; a representative analysis from single images is shown. Exposure to DON/DMSO significantly reduced de novo protein synthesis when compared to control populations of cells (**C**); data are shown from five replicate experiments and are expressed as mean + standard deviation (SD). Representative 20× magnified images from cells treated with DMSO (**D**) and DON/DMSO (**E**) are shown; green represents de novo protein synthesis and blue is a DAPI nuclear stain. **** indicates *p* < 0.0001.

An increased abundance in free amino acids in MDBK renal epithelial cells following DON exposure is suggestive of decreased protein synthesis. To investigate this, de novo protein synthesis was quantified in MDBK cells cultured in the presence or absence of DON using immunohistology. A significant reduction in the abundance of newly synthesized protein was observed in MDBK cells exposed to DON compared to the control cells. This included a 44% decrease in proteins in the cytoplasm and a 58% decrease in nuclei, when compared to controls (*p* < 0.0001 for both locations; Figure 4A–E). In the absence of DON, cells showed significantly greater levels of newly synthesized protein in the nuclei when compared to the cytoplasm (*p* < 0.0001); however, following DON treatment, this difference was abolished, and newly synthesized proteins were equally distributed between the cytoplasm and nuclei (Figure 4C).

### 2.4. A Reduction in MDBK Proliferation Was Observed after DON Treatment

**Figure 5:**
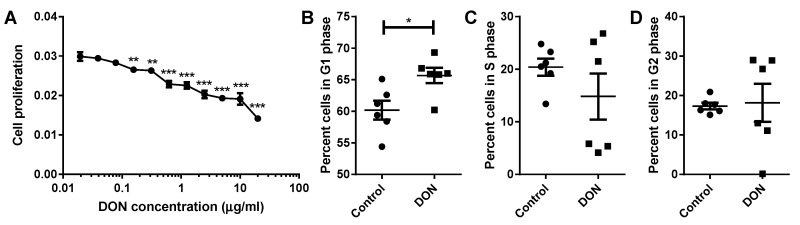
Cells treated with DON show reduced levels of proliferation, even at quite low concentrations of DON. Proliferation of MDBK cells was significantly reduced following exposure to concentrations of DON greater or equal to 0.16 µg/mL when compared to control cells (**A**). DON-treated cells accumulated in the G1 phase of the cell cycle (**B**) and failed to progress to S phase (**C**) and the subsequent G2 phase (**D**). Data are shown from six replicate experiments and are represented as mean ± SEM. * *p* < 0.05, ** *p* < 0.01, and *** *p* < 0.001.

To investigate the impact of decreased protein synthesis on renal epithelial cell turnover, MDBK cells were exposed to a range of concentrations of DON and proliferation was quantified. MDBK renal epithelial cells showed a significant decrease in cell proliferation following treatment with concentrations of DON greater or equal to 0.16 µg/mL when compared to control cells (0.16–0.31 µg/mL DON *p* < 0.01, 0.63–20 µg/mL DON *p* < 0.001; Figure 5A). Disruption to the normal renal epithelial cell proliferation rate was observed at markedly lower concentrations of DON than those which induce cell death; a significant decrease in cell proliferation was seen at concentrations of DON 14 times lower than the minimum required to induce significant cell death.

To further investigate the inhibitory effect of DON on renal epithelial cell proliferation, cell cycle analysis was carried out on MDBK cells cultured in the presence or absence of DON. Following exposure to DON, a significantly greater proportion of MDBK cells were in the G1 phase compared to control populations of cells (*p* < 0.05; Figure 5B). This indicates that the observed reduction in cell proliferation is due to renal epithelial cells arresting in the G1 phase of the cell cycle in the presence of DON.

## 3. Discussion

This study characterized the effects of the common mycotoxins DON, ZEN, FB1, and PA on bovine kidney epithelial cell cytotoxicity, metabolism, protein synthesis, and proliferation. While FB1 showed no cytotoxic effects and ZEN was weakly cytotoxic (>100 μg/mL), cytotoxic effects of PA and DON were observed at 1 and 2 μg/mL, respectively. However, at sub-toxic doses, only DON induced significant changes to intracellular metabolism. The responses observed likely stem from the various insults that DON imparts on the cell machinery: DON has previously been documented as a ribotoxin and inhibitor of protein synthesis in other cell types, as well as a disruptor of both cellular and mitochondrial membrane integrity [1,2]. These are discussed below in more detail.

### 3.1. DON Inhibits Protein Synthesis and Arrests the Cell Cycle in Bovine Renal Epithelial Cells

Inhibition of protein synthesis by DON and other tricothecenes has been well studied [27]. Specifically, DON inhibits RNA translation by one of four mechanisms: Interfering with the peptidyl transferase function of the ribosome, which impairs initiation and elongation [27,28]; inducing phosphorylation of the eukaryotic translation initiation factor 2-apha (eIF2-apha) [27,29]; promoting degradation of 28s ribosomal RNA (rRNA) [30]; and upregulating microRNAs (miRNAs) that complement ribosomal protein messenger RNA (mRNA) [27]. This in turn initiates a ribotoxic stress response, namely activation of the mitogen-activated protein kinases (MAPK) pathways, which coordinate survival responses to stress. Notably, they modulate growth, differentiation, and apoptosis [31]. These processes appear to be reflected in this study. DON significantly reduced both nuclear and cytoplasmic protein synthesis, resulting in an accumulation of free alpha amino acids and reduced cell proliferation, arresting it at the G1 phase, without causing apoptosis. This has also been observed in DON-exposed human and porcine intestinal epithelial cells, and chick embryo fibroblast DF-1 cells [32,33,34]. A proposed mechanism was via both the upregulation and mRNA stabilization of p21 via the extracellular signal-regulated kinases 1 and / MAPK (ERK1/2 MAPK) cascade [32], though cell cycle arrest in kidney epithelial cells has also been mediated by the p38–MAPK pathway [35]. An important characteristic of renal epithelial tissue is its ability to regenerate following injury, be it from impact or various disease states. However, DON-induced cell cycle arrest may seriously hamper its ability to do so.

### 3.2. DON Disrupts Both Cellular and Mitochondrial Lipid Membrane Integrity, Likely Triggering Both Osmoregulatory Responses and the Hypoxia-Induced Factor (HIF) Pathway in Bovine Kidney Cells

The ability of DON and other tricothecenes to induce lipid peroxidation via oxidative stress in a number of eukaryotic cell lines has been well documented [36,37]. This would disrupt the osmolarity of the cell (and incidentally also cell cycle checkpoint progression), to which kidney epithelial cells are particularly sensitive, due to their role in mammalian osmoregulation [38]. Consistently, a number of osmolytes were noted to change in the cells after exposure to DON. For example, choline was depleted while glycerophosphocholine (GPC) accumulated. This suggests either inhibition of choline synthesis to increase GPC abundance, an important osmolyte [39], or a simultaneous efflux/influx had occurred. Moreover, the cells sequestered the free amino acids that were available following protein synthesis inhibition (instead of diverting them to other pathways), which is another common response to hypertonicity [40]. Interestingly, the well-known organic osmolytes taurine and myo-inositol were depleted, which is rather a response to cell swelling (hypotonic conditions) [41,42]. However, unlike GPC, which is synthesized by the cells, both taurine and *myo*-inositol require active transport to retain their high concentrations in renal epithelial cells, which may be compromised upon exposure to DON.

The damage to lipid membrane integrity caused by DON extends to the mitochondria, where DON has been found to decrease mitochondrial membrane potential (MMP) [43] and thus also oxidative respiration. Mitochondrial reductases are capable of reducing MTT to formazan [44]. This reduction, reflecting the global metabolic activity, depends on dihydronicotinamide-adenine dinucleotide (phosphate) NAD(P)H-dependent oxidoreductase enzymes [45]. In this study, DON significantly altered the metabolic oxidoreductase activity in the mitochondria. Creatine, which recycles adenosine diphosphate (ADP) to adenosine triphosphate (ATP) was exhausted after exposure to DON. This disruption, and disruptions to the tricarboxylic acid (TCA) cycle, necessitates alternate modes of respiration. In this study, a marked increase in alanine and lactate, precursors and products of pyruvate, respectively, were observed when cells were exposed to DON. Such changes may reflect an increase in the anaerobic glycolysis pathway. In addition, increases in the beta oxidation of fatty acids were also apparent following DON exposure with β-hydroxybutyrate observed to increase (Figure 3). Depletion of beta-alanine in the cells was also noted, which can be diverted to fatty acid biosynthesis, via malonate and conversion to acetyl-CoA. Interestingly, lysine, which imports fatty acids to mitochondria, was also increased [46].

The various ion transport processes performed by renal tissue are ATP-dependent, and most of the O_2_ in the kidney is used for this. The kidney, however, does not possess the ability to alter partial pressure of oxygen (pO_2_) when it becomes low and is susceptible to hypoxic injury [47]. Renal cells thus employ a range of mechanisms to protect from hypoxic injury, in particular the induction of the hypoxia-inducible-factor (HIF) pathway [35,47], the sensors of which are 2-oxoglutarate-dependant oxygenases [48]. Notably, the HIF pathway induces and maintains anaerobic glycolysis and suppresses mitochondrial respiration and reactive oxygen species (ROS) generation. This is achieved by increased expression of glycolytic enzymes such as hexokinase, phosphofructokinase, aldolase, phosphoglycerate kinase 1, enolase, and lactate dehydrogenase and by blocking conversion of pyruvate to acetyl-CoA [47].

## 4. Conclusions

**Figure 6:**
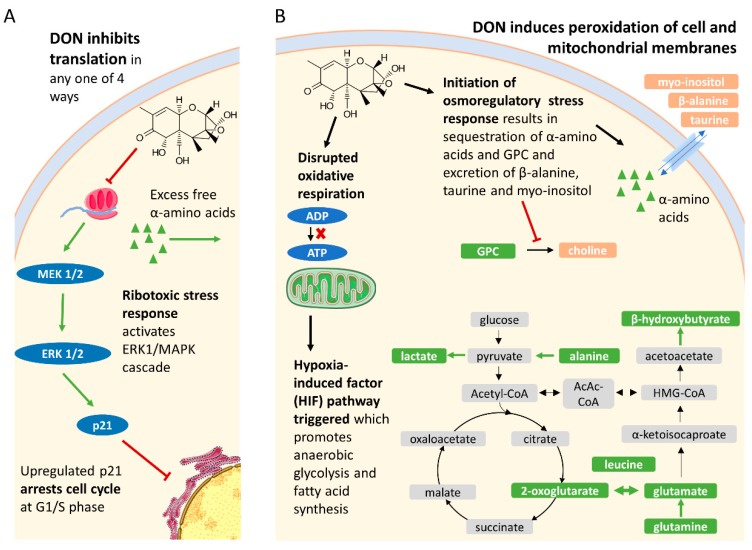
Model of DON toxicity to bovine renal epithelial cells. (**A**) DON inhibits protein translation, which triggers a ribotoxic stress response involving activation of the mitogen-activated protein kinases (MAPK) pathways, possibly the extracellular signal-regulated kinases 1 and / MAPK (ERK1/2 MAPK) cascade which upregulates p21 and arrests cell cycle at S phase. (**B**) DON peroxidates both mitochondrial and cell membranes. The damage to mitochondrial membrane integrity induces the hypoxia-induced- factor (HIF) pathway described in renal cells, which would switch oxidative respiration to anaerobic glycolysis and promote fatty acid production. Simultaneously, damage to the cell membrane would induce osmoregulatory responses which would sequester free amino acids (leucine, isoleucine, valine, glutamine, glutamate, lysine, and alanine) while excreting osmolytes beta-alanine, taurine, and myo-inositol. Green metabolites were increased with DON exposure and orange metabolite were depleted with DON. Image components from Smart Servier Medical Art (Creative Commons license CC BY 4.0).

This study demonstrates, for the first time, that low concentrations of DON induce hypoxic, hypertonic, and ribotoxic stresses in bovine renal epithelial cells, summarized in Figure 6. Nephrotoxicity would in turn significantly modulate hormonal, osmoregulatory, insulin regulatory, and excretory processes, hampering the ability of the animal to regulate nutrients and the elimination of DON and other toxins. This contributes to our understanding of how chronic low-level exposure to mycotoxins, such as DON, results in the symptoms associated with bovine mycotoxicosis and compromises productivity.

## 5. Materials and Methods

### 5.1. Determination of Cytotoxicity and Impact of Mycotoxins on Metabolic Activity of Bovine Kidney Epithelial Cells

The MDBK epithelial cell line (European Collection of Authenticated Cell Cultures (ECACC), Health Protection Agency Culture Collection, Salisbury, UK), derived from the kidney of an adult bovine (*Bos taurus*), was used to investigate the cytotoxic potential of mycotoxins and their effect on cell metabolic activity. Cells were cultured in Minimal Essential Media (MEM) supplemented with 10% fetal calf serum, 2 mM L-glutamine, 1% non-essential amino acids, and 100 U/mL penicillin/streptomycin (all Sigma, Poole, UK) in 96-well tissue culture plates at an initial density of 5 × 10^4^ cells/well. After 48 h of culture at 37 °C with 5% CO_2_, cells were treated with a range of doses of individual mycotoxins, as informed by results of a previous silage survey [17] (DON (0.01–20 µg/mL), ZEN (0.01–200 µg/mL), FB1 (0.001–20 µg/mL), and PA (0.01–20 μg/mL); all Sigma, Poole, UK) and prepared in supplemented MEM, with the addition of 0.05% dimethyl sulfoxide (DMSO) to solubilize mycotoxins. Cells exposed to 0.05% DMSO only served as controls. Following a further 24 h of culture, supernatants were harvested, and the cytotoxic effect of mycotoxins was quantified using a Cytotox 96 non-radioactive cytotoxicity assay kit (Promega, Southampton, UK), as per the manufacturer’s instructions. Metabolic activity was determined by thiazolyl blue tetrazolium bromide (MTT) assay. Media were replaced with 0.15 mg/mL MTT (Sigma, Poole, UK) prepared in MEM supplemented as described above. Cells were cultured for 30 min before the media was removed and cells were treated with DMSO to solubilize MTT formazan. Absorbance was read at 540 nm. Data were analyzed by one-way ANOVA with a Tukey post-hoc test (GraphPad Prism 7, GraphPad Software, San Diego, CA, USA).

### 5.2. ^1^H Nuclear Magnetic Resonance (NMR) Spectroscopy-Based Metabolic Phenotyping

MDBK cells were cultured in MEM, supplemented as above, at an initial density of 6 × 10^5^ cells/well in a 12-well plate. After 48 h of culture at 37 °C and 5% CO_2_, the media was replaced, and cells were treated with DON (1 µg/mL), ZEN (20 µg/mL), FB1 (0.5 µg/mL), and PAT (0.1 µg/mL) plus 0.5% DMSO. Mycotoxin concentrations were selected below the level which induced significant cytotoxicity (Figure 1). Following an additional 24 h of culture, the media were removed, and cells were washed twice with phosphate-buffered saline (PBS) before quenching with ice cold methanol. Cells were stored at −80 °C until further processing. Culture samples were placed on ice and 300 µL CHCl_3_/MeOH (2:1) solution (Honeywell, Seetze, Germany) was added. Samples were vortexed (30 s), 300 µL of H_2_O was added and samples were again vortexed (30 s) before centrifugation at 14,000× *g* for 10 min. The aqueous layer was sampled and, subsequently, freeze-dried. The lyophilized samples were reconstituted in 540 µL H_2_O and combined with 60 µL of phosphate buffer (1.5 M KH_2_PO_4_, 2 mM NaN_3_ and 1% trimethylsilylpropanoic acid (TSP) in D_2_O; Sigma-Aldrich, Steinhem, Germany). The mixture was vortexed, spun (14,000× *g*, 10 min), and 550 µL was transferred to 5 mm diameter NMR tubes (Bruker, Rheinstetten, Germany). Quality control samples were created by pooling portions of all samples and preparing them as described above.

The samples were analyzed using a 600 MHz Bruker Avance III spectrometer (Rheinstetten, Germany). ^1^H NMR spectra were acquired using a standard (1D) pulse sequence, using the first increment of the nuclear Overhauser effect (NOE) pulse sequence for water suppression. Raw spectra were phased, baseline corrected, and calibrated to TSP using Topspin 3.2 (Bruker Biospin, Rheinstetten, Germany). The spectra were then imported into MATLAB (Version R2014a; Mathworks Inc., USA) for further processing, namely removal of redundant peaks, manual alignment of peaks using in-house MATLAB scripts. The resulting spectra were analyzed using principal-component analysis and projection to latent structures-discriminant analysis (PLS-DA). For the PLS-DA model the metabolic profiles served as the X matrix and the dosing groups were used as the Y (response) vector (DMSO treated versus DMSO/DON treated). The predictive performance of the model (Q^2^Y) was calculated using a seven-fold cross-validation approach and the significance of the Q^2^Y value was determined through permutation testing (1000 permutations). A coefficients plot was generated from the PLS-DA model outputs. Here, the covariance of the peaks (metabolites) with sample class was plotted (i.e., positive peaks were more abundant in cells treated with DON while negative peaks were reduced in cells treated with DON) and the peaks were colored by their correlation to the predictive component.

### 5.3. De Novo Synthesis of Protein by Bovine Kidney Epithelial Cells Following DON Exposure

MDBK cells were cultured in MEM plus supplements in 96-well tissue culture plates at an initial density of 2.4 × 10^4^ cells/well. Cells were incubated at 37 °C with 5% CO_2_ for 24 h before the addition of 1 µg/mL DON plus 0.05% DMSO to test wells and 0.05% DMSO to control wells. Following a further 24 h of culture, de novo protein synthesis was quantified using a Click-iT HPG Alexa Fluor Protein Synthesis Assay Kit (Thermo Fisher Scientific, Waltham, MA, USA). Fluorescence was captured on a DMIRE2 microscope (Leica, Wetzlar, Germany) equipped with an ORCA-R2 camera (Hamamatsu Photonics, Hamamatsu City, Japan). Ten fields of view per well at 20× magnification were digitized using Leica MM AF software. Mean fluorescence intensity in the cytoplasm and nuclei was determined using ImageJ software (http://rsb.info.nih.gov/ij). Data were analyzed by two-way ANOVA with a Sidak’s multiple comparisons test (GraphPad Prism 7, GraphPad Software, San Diego, CA, USA).

### 5.4. Proliferation of Bovine Kidney Epithelial Cells Following Exposure to DON

MDBK cells were cultured in MEM, supplemented as previously described, in 96-well tissue culture plates at an initial density of 2.4 × 10^4^ cells/well for 48 h at 37 °C with 5% CO_2_. Media were replenished, and cells were treated with a range of doses of DON (0.02–20 µg/mL) plus 0.05% DMSO. At the same time, cells were labeled with bromodeoxyuridine (BrdU) to quantify proliferation. After 24 h of culture BrdU incorporation was quantified using the Cell Proliferation Biotrak ELISA system (GE Healthcare, Chalfont St Giles, UK), as per the manufacturer’s instructions. Data were analyzed by one-way ANOVA with a Tukey post-hoc test (GraphPad Prism 7, GraphPad Software, San Diego, CA, USA).

### 5.5. Cell Cycle Analysis of Bovine Kidney Epithelial Cells Exposed to DON

MDBK cells in MEM plus supplements were seeded into 12-well tissue culture plates at an initial density of 3 × 10^5^ cells/well and cultured for 24 h at 37 °C with 5% CO_2_ before the addition of DON (1 µg/mL) plus 0.05% DMSO to test wells and 0.05% DMSO to control wells. After a further 24 h of culture, the media were removed, cells were detached from the plate by the addition of trypsin-EDTA (0.05% trypsin, 0.02% EDTA; Sigma) and prepared for cell cycle analysis as previously described [49]. Cells were washed and fixed by the addition of 5 mL 95% ethanol. Following fixation, cells were centrifuged at 500× *g* for 5 min and the ethanol was removed. Cells were permeabilized by the addition of 1 mL 2N HCl plus 0.5% Triton X-100 and incubated at room temperature for 30 min. Cells were centrifuged as previously, the supernatant was discarded, the pellet was resuspended in 1 mL 0.1 M NaB_4_O_7_ (pH 8.5) and incubated at room temperature for 30 min. Cells were centrifuged again, resuspended in 500 µL propidium iodide (PI) and RNase solution (50 mg/mL propidium iodide (Sigma) and 0.25 mg/mL RNase A (Sigma)), and incubated at 4 °C overnight, protected from light. Cells were analyzed using a CytoFlex flow cytometer (Beckman Coulter, Brea, CA, USA) and FlowJo software. Cells were initially gated on forward and side scatter and doublets were excluded. The proportion of cells in each phase of the cell cycle was calculated using an algorithm contained within the FlowJo software (FlowJo LLC, Ashland, OR, USA). Data were analyzed by one-way ANOVA with a Tukey post-hoc test (GraphPad Prism 7, GraphPad Software, San Diego, CA, USA).
